# The Impact of Acute and Chronic Exercise on Immunoglobulins and Cytokines in Elderly: Insights From a Critical Review of the Literature

**DOI:** 10.3389/fimmu.2021.631873

**Published:** 2021-04-14

**Authors:** Maha Sellami, Nicola Luigi Bragazzi, Baha Aboghaba, Mohamed A. Elrayess

**Affiliations:** ^1^ Physical Education Department (PE), College of Education, Qatar University, Doha, Qatar; ^2^ Laboratory for Industrial and Applied Mathematics (LIAM), Department of Mathematics and Statistics, York University, Toronto, ON, Canada; ^3^ Department of Health Sciences (DISSAL), Postgraduate School of Public Health, University of Genoa, Genoa, Italy; ^4^ Program of Exercise Science, College of Health and Life Sciences, Hamad Bin Khalifa University, Doha, Qatar; ^5^ Biomedical Research Center, Qatar University, Doha, Qatar

**Keywords:** exercise, immunoglobulin, interleukin, cytokine, tumor necrosis factor alpha

## Abstract

The level of immunoglobulins and cytokines changes with an ageing immune system. This review summarizes findings from studies that have examined the impact of acute and chronic exercise on immunoglobulins and cytokines in the elderly. Our literature analysis revealed that acute endurance exercise resulted in increased secretory salivary immunoglobulin A (SIgA), while acute bouts of muscle strengthening exercise (i.e., isokinetic, eccentric, knee extensor exercise) increased plasma/muscle interleukin (IL)-6, IL-8 and tumor necrosis factor alpha (TNF-α) levels. Chronic exercise in the form of short-term endurance training (i.e., 12-16 weeks) and long-term combined endurance and resistance training (i.e., 6-12 months) induced increases in salivary SIgA concentration. We additionally identified that short-term endurance training at moderate intensities and the combination of endurance, strength, balance, and flexibility training increase plasma IL-10 and reduce plasma IL-6 and TNF-α in healthy elderly adults and male patients with chronic heart failure. Strength training for 6-12 weeks did not alter plasma IL-1β, IL-2, IL-6 and TNF-α concentration in healthy elderly adults and patients with chronic-degenerative diseases, while 12 weeks of resistance training decreased muscle TNF-α mRNA in frail elderly individuals. Short-term (i.e., 10-24 weeks) moderate- to high-intensity strength training reduced LPS–IL-6, LPS, IL-1β, LPS–TNF-α and circulating concentrations of TNF-α and increased IL-10 in healthy elderly women and older people with cognitive impairment, respectively. In conclusion, it appears that acute bouts of endurance exercise and short-term chronic exercise training exercise are appropriate methods to enhance mucosal immune function, reduce systemic markers of inflammation, and promote anti-inflammatory processes in elderly individuals.

## Introduction

Aging represents a complex, multi-step, multi-factorial process involving an accumulation of changes affecting different functions and systems (such as the immune, the metabolic, the endocrine, and the cardiovascular systems). These changes may be associated with increased morbidity and mortality (1–3) and can be either caused by biological aging or represent compensatory mechanisms following age-related changes (4, 5).

Aging has also been associated with a decline in cardio-respiratory fitness, which in turn affects performance capacity, including aerobic performance (e.g., 6-min-walk test) (6, 7). Moreover, there is evidence for muscle weakness (i.e., dynapenia, sarcopenia) in old age also (8, 9). These deteriorations are caused by the complex interplay of various neural, metabolic, hormonal, muscular, and environmental factors (10).

With advancing age, several alterations (both morphological and functional) in the immune system due to immunosenescence (termed also as immunopause or immune dysregulation) have been reported (5). Important drivers of these changes could be i) the thymic involution, resulting in ii) a reduced number of lymphoid precursor B- and T-cells, a less effective adaptive immune system with an impaired lymphocyte proliferative capacity, depressed phagocytic and chemotactic responses, and a subsequent (quantitative and qualitative) decrease in the immune response. All this leads to iii) an accumulation of pro-inflammatory cell populations, including an increase in the number of natural killer (NK) cells, and a higher secretion of cytokines in adipose tissue, leading to an increased, generalized chronic activation of the innate immune system resulting into a low-grade, chronic inflammatory and oxidation background (known as inflammaging/oxi-inflammaging), and iv) an increased production and release of auto-antibodies (11–13). In fact, a recent study found that the elderly compared with young individuals showed higher levels of interleukin type 6 (IL-6), IL-1, tumor necrosis factor alpha (TNF-α), and C reactive protein (CRP), which were associated with a higher risk of morbidity (14).

Exercise can modulate and counteract such changes, reducing inflammation levels, preserving thymic mass, improving immunosurveillance, and protecting against psychological stress (15). For instance, Calle and Fernandez (16) examined how resistance training can help minimize the effects of inflammatory-related diseases in obese individuals at risk of sustaining low-grade inflammatory disease. Authors reported that long-term resistance training appeared to ameliorate inflammation. Resistance training (like weight training, weight machine or isometric exercise) may result in increased muscle strength. It is believed to counteract or, at least partially, mitigate the insurgence and the burden generated by metabolic and cardiovascular disorder.

Besides resistance training, endurance training, essential for sports disciplines such as running, marathon, long-distance swimming or mountain climbing, includes the regular practice of low-moderate intensity exercise, generally strengthening the aerobic system and cardio-respiratory fitness, along with building muscles. Sprint training includes high-intensity, high-speed short bouts of exercise (such as 10-, 100- or 800-m running and races) and results in burning fat, enhancing the endocrinological functioning and system, along with enhancing muscle mass.

However, the specific effects of the different types of exercises/physical activity (in terms of intensity, mode, and duration) on the inflammatory cytokines, including TNF-α, are not yet clear. A comprehensive, updated synthesis of the literature on this topic was therefore carried out. The present review may also help practitioners to design and adopt appropriate training/exercise programs for these specific populations. As such, the aim of this study was to describe and summarize the impact of acute and chronic physical exercise on immunoglobulins, with a focus on salivary secretory immunoglobulin A (SIgA), and cytokines in healthy old adults and patients with chronic disorders.

## The Immune System

### Immunoglobulins and Cytokines

B-cells produce immunoglobulins that can help in the identification of infectious agents and fight against bacteria or viruses. Immunoglobulins, also known as antibodies, are glycoprotein molecules produced by plasma cells (effector B-cells). There exist five major antibody classes including: IgA, IgD, IgE, IgG and IgM. This classification reflects differences in the amino acid sequence in the constant region (Fc) of the antibody heavy chains. IgG and IgA are further grouped into subclasses (e.g., in human IgG1, IgG2, IgG3, IgG4, IgA1 and IgA2) based on additional small differences in the amino acid heavy chain sequences (15–18).

These glycoproteins act as a critical part of the immune response by specifically recognizing and binding to antigens, mostly to protein antigens but also to some carbohydrates, either infectious or not. Binding to antigens of bacteria, viruses or other pathogens may result in their destruction, as well as in other biological events. For instance, SIgA may bind to microbiota bacteria and do not directly destroy them but prevent their attachment to the *epithelia* (a process known as “immune exclusion”). This phenomenon consists in the agglutination of polyvalent antigens and their subsequent crosslinking, trapping within the mucus layer *via* the oligosaccharide chains of the immunoglobulins, and peristaltic clearing. Immunoglobulins may also help in antigen neutralization (for virus mostly), uptake for antigen presentation, and opsonization for phagocytosis.

IgG molecules possess heavy chains known as γ-chains, whereas IgM has μ-chains, IgA has α-chains, IgE has ϵ-chains, and IgD has δ-chains.

Free light-chains can be usually found in inflammatory and autoimmune conditions, often associated with ageing. They were primarily measured for diagnosing blood cancers such as myeloma, but now free light chains are thought to have direct anti-microbial action. Furthermore, recently, studies have shown that free light-chains may serve as a useful biomarker for monitoring the effectiveness of exercise interventions in healthy and clinical populations. Immunoglobulins can be measured in saliva and secretory immunoglobulin A or sIgA represents the most common form of IgA circulating in the human body (>85% of the total IgA levels). SIgA is secreted by B-lymphocyte cells in the *lamina propria* of all mucosal tissues, mostly in the intestine. For operational reasons, SIgA populating the salivary glands is sampled and measured. Its concentration tends to decrease with aging (19–21), due to a reduced salivary follow and secretion rate.

Cytokines constitute a large group of proteins, peptides or glycoproteins that are secreted by specific cells of the immune system and other secretory organs and contribute to cell signaling. The term cytokines may represent chemokines, interferons, interleukins, and lymphokines (22). These proteins are produced by several cells such as macrophages, B- and T-lymphocytes, endothelial cells, fibroblasts, and various stromal cells, including muscles. Myokines are signaling proteins produced by muscle cells that have been extensively implicated in aging (23–25). They have different effects on organs (liver, bone, pancreas) and exert various functions such as the regulation of muscle hypertrophy and myogenesis (26, 27) or the modulation of cellular stress (28).

Recently, cytokines and myokines have been proposed to belong to a superfamily of molecules termed as exerkines, which include also nucleic acids, peptides and proteins, and metabolites (29). Adipokines are also important molecules that are classified as exerkines (30) and they are critical in the elderly since adipocytes increase in number with ageing. Some authors (31–33) also include hepatokines (produced by the liver), osteocalcin and other bone secreted factor in the list of exerkines, emphasizing the complex crosstalk among different tissues and organs (muscle, bone, liver and adipose tissue) (34–36).

## Acute Bouts of Exercise

### Salivary Secretory Immunoglobulin-A (SIgA) and the Effect of Exercise

Concerning the effect of exercise, some studies have shown that acute bout of exercise, including incremental all-out treadmill exercises, may decrease salivary IgA in young athletes (37–46), whereas other studies have found no association between SIgA and exercise (47–62). Very few studies reported, instead, an increase of SIgA (63–66).

These inconsistent findings could depend on a variety of factors: lifestyle behaviors and different nutritional status of the studied populations (for instance, plasma glutamine concentration, which could mediate the levels of SIgA before and after exercise), the study design (cross-sectional *versus* longitudinal), technical variables including sample collection timing, sample transport, storage and pre-processing, and assay employed (enzyme immune assay, lateral flow assay, or point-of-care testing, which can vary in terms of reproducibility, reliability, sensitivity and specificity), sport-related variables (time between exercise and sample collection, type of training program, and type of exercise, in terms of intensity, modality and frequency), as well as individual variation, biological and circadian rhythms, and psychological factors (type of personality, and exposure to stressors, among others) (67).

Aging exerts subtle and complex effects on SIgA levels: mean SIgA levels tend to increase with age up to 60 years, and then slightly decrease (68). Concerning the effects of exercise among the elderly, Sakamoto and collaborators (69) recruited 92 community‐dwelling old women aged over 75 years, living in a rural area, who periodically performed approximately 20 min of low intensity exercise. In comparison with before exercise, saliva flow, SIgA concentration and secretion rate were significantly increased. Neves and coauthors (70) examined the acute effects of resistance exercise sessions performed at different intensities (50 *versus* 80% of one-repetition maximum) on SIgA absolute concentrations in a sample of 15 elderly women, aged 67.5 ± 3.9 years, performing two sets of 13 repetitions at 50% 1RM and two sets of eight repetitions at 80% 1RM. Resistance exercise sessions induced significant elevation in SIgA levels, compared to control session. Teixeira and coworkers (71) analyzed the influence of a 19-week exercise program on SIgA. Thirty-three subjects aged 68-95 years old participated and were distributed into 2 groups: 15 subjects performed aerobic endurance and lower and upper body strength exercise that included low-impact rhythmic work sequences with music, 3 times a week, and 18 remained sedentary. For the exercising group sIgA levels were higher after the 19-week exercise program with no changes for the control group.

These findings seem to suggest that exercise had mixed effects in youth, decreasing or not altering salivary IgA in young individuals, with few reports of increased values, while acute resistance and endurance exercise tended to increase salivary IgA levels in elderly people. Although the direct mechanisms underpinning exercise-induced changes in salivary IgA are unclear, neuroendocrine factors could be involved (e.g. cortisol) (72–74). In conclusion, SIgA is one of the most important mechanisms responsible for the defense against microbial invasion and health benefits that are associated with acute endurance and resistance exercise, which seems to counteract or at least partially mitigate the effects of aging.

### Saliva Free Light-Chains and the Effect of Exercise

Free light-chains are a major biomarker of malignancies, including autoimmune disorders and immunological impairments (75, 76).

Saliva free light-chains concentrations and secretion rates were measured in a sample of 88 young subjects, aged 18-36 years, and in a sample of 53 older adults, aged 60-80 years (75). While young adults completed a constant work-rate cycling exercise trial at 60% VO_2max_ or a 1 h cycling time trial, older adults completed an incremental sub-maximal treadmill walking exercise test to 75% HR_max_. Saliva free light-chains levels were higher in older subjects: 3.91 [95%CI 0.75-19.65] mg/L and 1.00 [95%CI 0.02-4.50] mg/L in older adults *versus* 0.45 [95%CI 0.004- 3.45) mg/L and 0.30 [95%CI 0.08-1.54] mg/L in young adults, for kappa and lambda, respectively

## Immunoglobulins Production and Release During Exercise

Besides sIgA, other immunoglobulins have been relatively overlooked in the existing scholarly literature. Acute sub-maximal exercise appears to primarily affect serum IgM levels in young athletes (77). Preliminary data indicated increases in IgM levels (19), which have to be confirmed in future studies in elderly individuals. Various mechanisms of stimulation such as the modulation by hormones and the release of interleukins have been proposed to explain the exercise-induced effect on IgM in seniors (19).

## Free Light-Chains Production and Release During Exercise

Serum kappa and lambda free light-chain responses to acute sub-maximal exercise were measured in 45 healthy older adults (aged ≥60 years) who were either sedentary, physically active or endurance trained (76). It was found that the endurance trained group had significantly lower levels of kappa and lambda serum free light-chains compared with physically active or sedentary elderly adults, without any significant difference in whole immunoglobulin levels among groups. These findings seem to suggest that endurance exercise may reduce serum kappa and lambda free light-chains production and release in elderly people (76).

## Cytokine and Myokine Serum Production and Release During Exercise

The existing literature on the topic reports contrasting findings: some scholars describe age as a mediator of the relationship between exercise and myokines levels, whilst others fail to replicate these results. For instance, Gmiat and coauthors (78) evaluated the effect of a single bout of high-intensity circuit training using body weight as resistance on myokines concentration (IL-6, irisin, IL-10, TNF-α) in a sample of fourteen healthy, non-active women assigned to a young or middle-aged group. Age impacted on myokines concentration 1 hour after the high-intensity exercise. The effect on irisin concentration was moderate and trivial. Changes in IL-10, IL-16 and TNF-α were moderate in the middle-aged group, whilst they were small-to-moderate in the young-aged group.

On the other hand, a recent systematic review of the literature and meta-analysis (79) computed a post-exercise average increase in elevated irisin concentration induced by an acute bout of exercise of 15.0 [95%CI 10.8-19.3], without any impact of age.

Pedersen et al. (28, 29) and Hamada et al. (80) found that acute eccentric exercise increased the skeletal muscle mRNA levels of TNF-α, IL-1 in adults aged 66–78 years. Results from another study suggest that resistance training induces mRNA expression of IL-1β, IL-2, IL-5, IL-6, IL-8, IL-10, and TNF-α in muscle tissue without its increment on plasma in recreationally active older women (81).

A previous study reported that treadmill exercise for a maximum of 5 min slightly increases systemic TNF-α, but not IL-6 concentrations in healthy older men (82). Accordingly, the IL-6 release from working muscles after 60 min of workout is preserved in healthy, 70-year-old men (83) when a two-leg knee extensor exercise is performed without muscle damage. Reihmane et al. (84) reported that 45 min of two-leg dynamic knee extensor exercise at 19.5 ± 0.9 W increases IL-6 release from rest to 30 min of exercise but was not higher than the resting level after 45 min of exercise. In addition, after isokinetic exercise, the expression of MCP-1, IL-8 and IL-6 (pro-inflammatory) increased substantially while, the expression of the anti-inflammatory cytokines IL-4, IL28 and IL-13 increased only slightly (or not at all) after exercise (85). In contrast, IL-6 mRNA is decreased in elderly men who perform downhill running, and the increase in systemic IL-6 levels is modest in elderly men who perform eccentric leg exercise (86) compared with young controls.

The effects of age on the kinetics of post-exercise cytokine levels were also analyzed. Some studies have observed greater cytokine expression within skeletal muscle of older individuals (87, 88). In contrast, other studies have failed to report significant differences in cytokine expression in skeletal muscle between young and elderly individuals at rest (87–89), including TNF-α expression (90).

More studies in this area are needed to confirm the discordant changes in cytokine transcriptional changes between young and old individuals and to elucidate the roles of myokines in the systemic perturbations to acute exercise. In addition, some authors studied that effect of exercise intensity on cytokine levels. They identified that maximal exercise tests on a treadmill, consisting of walking at 3.4 km h^-1^0% inclination for five minutes, followed by an intensive ramp protocol, whereby the treadmill speed increased by 0.1 km h-1every 4 sec (i.e., a 1.5 km h-1increase each minute) until exhaustion, similarly decreased TNF-α in both higher-volume (HVG,~480 min/week) and lower-volume groups (LVG, ~240 min/week), whereas CRP increased differentially (+60% LVG; +24% HVG; p<0.05).

A systematic review published by de Salles et al. (91) analyzed the effectiveness of resistance training studies on cytokines in a broad array of populations: men and women; young, adult and older individuals; overweight and obese and, finally, patients with multiple sclerosis, and subjects infected with human immunodeficiency virus (HIV). Authors synthesized 17 longitudinal clinical studies and found evidence that resistance training leads to increased level of adiponectin, and to decreased level of leptin and CRP. Training was effective in reducing CRP level in obese individuals and older adults, whilst had no impact on TNF-α concentration. Variables such as intensity (greater than 80% of one repetition maximum) and duration (more than 16 weeks) seem to moderate the response.

Overall, by reviewing the evidence, the current review shows that IL-6, IL-8 and TNF-α increase during acute strength exercise (dynamic knee extensor, isokinetic, eccentric) lasting less than 45 min. For instance, skeletal muscle is a major source of IL-6, IL-8 and TNF-α during strength exercise, as mRNA levels, and protein levels increase largely within muscle fibers, and the IL-6 release from working muscles can largely account for systemic increases during physical activity (i.e., strength exercise).

However, as stated by Bruunsgaard (92), who analyzed the effects of exercise on various inflammatory markers in healthy and patient individuals, due to the limited available information in the literature and the lack of a review article on this topic, there is a need to summarize the few available studies on acute and chronic effects of physical exercise on immunoglobulins and cytokines in healthy elderly adults and patients with diseases linked to ageing and lifestyle.

As for immunoglobulins, these contrasting results could be due to various factors, reflecting differences in lifestyles of the recruited population, in the methodology adopted, and, above all, in the training/exercise protocol studied. Individual variability and circadian effects may play a role too (93). Cytokines levels can oscillate within a timeframe of 24 hours, displaying variations on a daily basis, mainly driven by the internal time keeping system known as circadian clock (93). Circadian and other biological rhythms impact myokines production and release, too (94): according to an emerging and accumulating body of empiric evidence, the basal secretion of myokines by human skeletal myotubes is finely tuned by a high-amplitude cell-autonomous, oscillating circadian clock. Furthermore, stressors and other environmental stimuli may exert effects on cytokines and myokines release (95). Interactions between muscle, bone, brain are yet to be elucidated and fully understood (95).

## Impact of Chronic Exercise and Training on Immunoglobulins and Cytokines in the Elderly

### Endurance Training and Salivary Immunoglobulin-A and Immunoglobulins

A limited number of investigations has analyzed the effect of endurance training on SIgA in elderly individuals. For instance, active daily walking exercise training preserves mucosal immunity. Shimizu et al. (96, 97) recruited a sample of 51 males and 74 females, who took part into regular exercise sessions, 5 days a week for 6 months. Authors found a significant increase in SIgA level after training (p<0.05). Similarly, 30-minute active walking exercise training with an intensity of 80% ventilatory threshold for 12 weeks (98) led to elevated SIgA secretion rates in a sample of thirty elderly people, 8 men and 22 women, aged 66.7 ± 7.4 years (from 43.4 ± 28.4 to 66.8 ± 45.0 μg/min, p=0.003). In particular, stratifying according to the characteristics of the sample, the increase was marked both in participants who were less than 64-year-old (from 44.9 ± 35.0 to 70.9 ± 44.6 μg/min, p=0.021) and 65-85-year-old (42.8 ± 26.6 to 65.4 ± 46.0 μg/min, p=0.025) elderly subjects. The change was found in females (39.0 ± 29.6 to 59.0 ± 40.2 μg/min, p=0.015) whilst no statistically significant changes could be detected in male individuals (55.5 ± 22.3 μg/min at the baseline and 88.5 ± 53.0 after 3 months, p=0.104).

These findings were replicated by a 16-week home-based walking program, which was found to increase resting SIgA secretion rate (+37.4%; p<0.05) in 32 elderly healthy postmenopausal women. However, immediately after the acute bout of exercise, the secretion rate of SIgA and the saliva flow rate were significantly reduced (-32.3%, p<0.05, and -29.3%, p<0.05, respectively) (99). Dudhrajh (100) evaluated the impact of a 12-week group exercise program on salivary biomarkers of mucosal immunity in a sample of 95 elderly individuals aged between 60-86 years, recruited from five aged care facilities in South Africa. Subjects were allocated to two groups, which underwent a twice/week program (n=40) and a three times/week program (n=45). Increases in SIgA secretion rate were significant in both groups with small-to-moderate effect sizes (twice/week p=0.07, Cohen’s *d*=0.44; three times/week p=0.09, Cohen’s *d*=0.34).

Hwang et al. (101) studied a sample of 12 older women divided into a Pilates group (PG, n=6) and a control group (n=6). After the three-month Pilates exercise program, salivary flow rate was significantly higher in the PG than in the controls, in particular 30 min after acute high-intensity exercise.

All this, taken together, suggests that short-term endurance training may contribute to reinforcing the immune system in elderly individuals, by positively impacting mucosal immunity.

### Endurance Training and Cytokines

Exercise training can induce significant changes in cytokine secretion and signaling processes. Shinkai et al. (102, 103) reported that a higher concentration of IL-2 (p=0.021), IFN-gamma (p=0.015) and IL-4 (p=0.012) were found in a sample of 17 habitual male runners aged 63.8 ± 3.3 years compared to control group.

Rhind et al. (104) and Ogawa et al. (105, 106) also reported that elderly women who had consistently walked for one and a half hours once a week for 4 years had a higher level of IL-2 compared to untrained women.

Drela et al. (107) found that the effect of 24 months of aerobic exercise training practiced by older women (aged 62-86 years) could induce an increment of the production of IL-2, but it did not cause changes in the gene expression of IL-4 and IFN-γ.

Gueldner et al. (108) recruited a sample of 46 independently dwelling, ambulatory and mentally alert women aged 60-98 years, to explore the percentage of CD25 mitogen stimulated lymphocytes. Authors found a higher surface expression of IL-2 alpha chain receptor CD25 on T-cells from when stimulated with mitogen *in vitro* in the active group (n=25) *versus* the inactive group (n=21).

In another study of 22 male sedentary individuals aged 71.27 ± 0.82 years, Santos et al. (109) explored the effect of moderate exercise training consisting of running for 60 min/day, 3 days/week over 24 weeks at a work rate equivalent to their ventilatory aerobic threshold. Authors reported a significant decrease in TNF-α (43%) and IL-6 (37%) levels, an increase in IL-10 (27%) and non-significant changes in IL-1 and CRP. However, regarding the effect of endurance training in elderly patients, it has been shown that six months of endurance training may reduce the expression of TNF-α (from 1.9 ± 0.4 to 1.2 ± 0.3 relative U, p<0.05), IL-6 (from 71.3 ± 16.5 to 41.3± 8.8 relative U, p<0.05) and IL-1β (from 2.7 ± 1.1 to 1.4 ± 0.6 relative U, p=0.02) in skeletal muscle of chronic heart failure patients while systemic levels of these cytokines were unchanged (110).

Accordingly, 12 weeks of aerobic exercise reduced TNF-α concentrations (111, 112). Furthermore, 12 weeks of aerobic exercise training at 70–80% of individual maximal heart rate consisting of 45 min sessions of continuous aerobic exercise on a treadmill, stationary bicycle, arm bicycle, rowing machine, or a combination of these activities resulted in a significant reduction of all pro-inflammatory cytokines, CRP, IL-1, IL-6, and INF-gamma, as well as a significant increase in the anti-inflammatory, cytokine IL-10 in elderly (64 ± 7.1 years) coronary heart disease patients (113). Additionally, Lima et al. (114) reported that 10 weeks of endurance training reduce plasma IL-6 levels and maintain TNF-α concentration in hypertensive older adults.

In contrast, Pilch et al. (115) reported that regular endurance training (high-low) for 3 times a week over 12 weeks induces a significant decrease in the serum IL-1β (from 2.56 ± 0.3 to 1.17 ± 0.2 pg/ml, Δ=-1.39 ± 0.5 pg/ml, p<0.05) and an increase in the serum IL-6 (from 36.3 ± 5.9 to 45.8 ± 6.5 pg/ml, Δ=9.5 ± 2.5 pg/ml, p<0.05) in 15 non–smoking middle-aged women (42-47 years).

Finally, based on the previous studies, it seems that moderate- to high-intensity endurance training can reduce IL-6 and TNF-α, and increase IL-10 in elderly healthy and patient with chronic heart failure male individuals (116–121).

## Strength Training

### ST and Salivary Immunoglobulin-A

Few studies have focused on the impact of chronic resistance training on SIgA concentration among old subjects. For instance, Ahn and Kim (122) investigated the effect of a resistance exercise program using elastic bands (frequency of 3 times/week, 60 min/day, for 4 months) on sIgA level. Twenty-two elderly women were divided into an exercise group (77.91 ± 1.41 years) and a control group (78.73 ± 1.51 years). Levels of sIgA tended to decrease following the exercise program, even though not reaching the statistical threshold. Moderate-intensity resistance training over 12 weeks did not alter SIgA in sedentary low active elderly people (123). Thus, SIgA levels remained unchanged after 6 months of resistance training in older adults (124). Therefore, 12 weeks and 6 months of resistance training may not be enough to stimulate elevation in salivary SIgA levels.

### ST and Cytokines

The influence of exercise on circulating levels of cytokines has been described as decreased, elevated or unchanged. Many studies investigated the effect of resistance training on cytokines.

Rall et al. (116) found that this intervention has no effect on IL-1β, IL-2, IL-6 and TNF-α in older adult subjects. Accordingly, Bruunsgaard et al. (117) showed no significant changes in TNF-α, IL-6 and sTNFR-I after 12 weeks of resistance training in frail elderly people (86-95 years). Accordingly, 6 weeks of cycle ergometry reduced sTNFR-II concentrations and maintained TNF-α and IL-6 in elderly patient with chronic heart failure (118).

In contrast, 3 months of resistance exercise decreased both muscle TNF-α mRNA and protein levels in frail elderly individuals (119). Many physical activity parameters participated in variable results on responsive cytokines to exercise training such as intensity, duration, and type of exercise.

For instance, Calle and Fernandez (16) reported that resistance training alters cytokines responses dependant on exercise intensity and duration in men.

Evidence from epidemiologic studies in older adults reported that greater levels of physical fitness are associated with lower circulating levels of several inflammatory biomarkers, such as, IL-6, TNF-α, and CRP (120, 121).

Based on the available studies, it seems that short-volume (i.e., 6-12 weeks) resistance training did not alter IL-1β, IL-2, IL-6 and TNF-α concentrations in elderly healthy and patient with chronic heart failure, while 12 weeks of resistance training decreased muscle TNF-α mRNA in frail elderly individuals.

On the other hand , 12 weeks of strength training involved low-intensity resistance exercise decreased plasma concentrations of CRP, SAA but, maintained IL-6, TNF-α, MCP-1 levels, after the training program in healthy elderly women aged 85.0 ± 4.5 years (65, 66). Accordingly, 28 weeks of strength training can exert anti-inflammatory effects in older people, resulting into an increase in IL-10 levels occurring conjunctly with a slight decrease in the TNF- α/IL-10 ratio and maintenance of TNF-α levels in 33 older women with cognitive impairment, aged 82.7 ± 5.7 years (125).

In contrast, 10 weeks of strength training at eight-repetition maximum significantly reduced LPS–IL-6, LPS–IL-1β, LPS–TNF-α and circulating concentrations of TNF-α in elderly women aged 72 ± 6.1 years (126).

In summary, short-term moderate- to high-intensity strength training reduced LPS–IL-6, LPS–IL-1β, LPS–TNF-α and circulating concentrations of TNF-α, while, low-intensity strength training did not alter the above-mentioned biomarkers in healthy elderly women. Furthermore, strength training lasting 28 weeks increased IL-10 levels and slight maintained TNF-α levels in older women with cognitive impairment. It seems that strength training created an anti-inflammatory environment and better inflammatory balance in older people with cognitive impairment.

Tibana et al. (127) assessed the effect of 16 weeks of resistance training (three sets of 10 exercises, 6-12 repetitions maximum and 1-min and 30-s rest intervals between sets and exercises, respectively, 2 sessions per week) on irisin level in a sample of 49 older women with and without obesity aged 61-68 years. Circulating irisin decreased in the non-obese group compared with pre-intervention and obese group (p=0.01 and p=0.04, respectively).

To summarize, with a focus on randomized controlled studies, a recent systematic review and meta-analysis (128) has shown that resistance training can reduce CRP in older adults (standard mean difference or SMD=-0.61 [95%CI -0.83 to -0.31], p<0.001) and IL-6 (SMD=-0.19 [95%CI -0.42 to 0.02], p=0.07, statistically borderline significant), whilst no changes in TNF-α level could be detected. Moderators of CRP and TNF-α changes were found to be muscle mass, as well as a higher number of exercises (>8), a higher weekly frequency (3 times/week) and longer durations than 12 weeks.

Kukuljan (129), Peake et al. (130) and Dalla Via et al. (131) recruited a sample of 180 men aged 50-79 years who participated in an 18-month program of progressive resistance training plus weight-bearing impact exercise (3 day/week). Serum IL-6 decreased and was 29% lower [95%CI -62 to 0].

## Sprint Training and Immunoglobulins and Cytokines

Limited investigations have analyzed the effect of sprint training on immunoglobulins as well as on cytokines. The few available studies have investigated in general the effect of this type of exercise training on cytokine and inflammation status in young (~20-30 yr) or middle aged (40 yr) subjects, while no studies were performed on older/elderly (>65 yr).

For example, Hovanloo et al. (132) found identical changes in inflammatory markers following sprint interval training and endurance interval training in young individuals. In addition, Davison (133) found that nine males who engaged in one session of sprint training did not have any changes in salivary SIgA concentration and secretion rate. In addition, Allen et al. (69) found no changes in TNF-α levels after high-intensity interval training (30 s sprint, 4-5 min passive recovery) or prolonged intermittent sprint training (10 s sprint, 2-3 min moderate exercise) in a sample of 55 sedentary, middle-aged individuals (mean age 49.2 ± 6.1 years). Participants underwent three training sessions per week for 9 weeks on a cycle ergometer.

Therefore, future investigations studying the effect of sprint training in cytokines in healthy and patient elderly are urgently needed.

## Combined Training and Immunoglobulins and Cytokines

Akimoto et al. (134) investigated the effect of resistance and moderate endurance training on SIgA. Forty-five elderly participants, 18 men, 27 women, aged 64.9 ± 8.4 years, performed 120 minutes’ resistance (60 minutes) and moderate endurance (60 minutes) training per week for 12 months. They found that the concentration and secretion rate of SIgA increased during 12 months of training in elderly individuals. Thus, 5 times per week for 6 month of moderate endurance and resistance training increased SIgA in sedentary elderly people (60-82 years). In fact, long-term combined resistance and endurance training (i.e., 6-12 months) seems to enhance mucosal immune function in elderly individuals.

Stewart et al. (135) failed to reveal an effect of 12 weeks of combined aerobic/resistance training on resting, fasting plasma IL-6, TNF-α, or IL-1β concentration in healthy elderly men and women. They reported that young subjects had higher plasma TNF-α concentrations compared with the older counterparts at baseline and after the intervention period. Accordingly, Lima et al. (74) reported no significant changes in plasma IL-6 and TNF-α after 10 weeks of combined aerobic/resistance training with duration of 30 sessions and a frequency of 3 times per week in hypertensive older adults. Furthermore, the combination of aerobic, strength, balance, and flexibility exercises intervention of moderate intensity resulted in a 32% reduction in CRP and a 16% reduction in IL-6 by after 12 months in elderly (70-89 years), non-disabled, community-dwelling men and women at risk for physical disability (136). Beavers et al. (137) reported that 6 months of combined training reduced only soluble TNF receptor class II (sTNFRII), IL-6 and IL-8 in elderly healthy men and women. In contrast, the same authors (138) reported that the combination of walking and interactive, group-mediated, behavioral focused sessions did not alter IL-6 soluble receptor, IL-18, and sTNFRII in older, overweight and obese community dwelling men and women at risk for cardiovascular disease.

This contradiction may be due the differences of type of exercises and the populations involved. In addition, 16 weeks of combined aerobic and resistance exercise training decreased both TNF receptors (but not TNF-α itself) in patients with chronic heart failure (139). Finally, it seems that the combination of aerobic, strength, balance, and flexibility exercises based interventions over a long term may be able to reduce CRP, sTNFRII, IL-6, IL-8 and TNF receptors in both older healthy subjects and patients with chronic heart failure.

Lima et al. (74) reported that after 10 weeks of training, no significant differences between aerobic training and combined aerobic/resistance training in IL-6 F and TNF-α in hypertensive older adults.

A number of limitations affecting the variations of SIgA and cytokines during acute and chronic exercise should be properly acknowledged. Different modes of training interventions are obvious reasons for discrepancies, e.g., endurance training vs. resistance training; differences in the intensity of exercise; and the time duration of the single bout of exercise, as well as the training volume. In addition, a large interpersonal variability in peripheral inflammatory markers in terms of parameter like salivary flow rate, circadian rhythm, menstrual cycle, or oral health status (which sometimes are not taken into account and corrected for) (11, 140), together with a considerable coefficient of variability in high sensitivity cytokine assays make power problems common. Additional studies are needed to assess the effects of different modes and intensities of exercise on inflammation.

## Conclusion

Individuals may exhibit a weakened immune system that does not respond effectively to various external stimuli due to their advanced age, chronic pathology and/or genetic factors. Therefore, it is necessary to introduce non-therapeutic interventions such as regular physical activity to boost their immune response and improve their overall health. Exercise exerts a wide range of effects on immune system (141). In this context, it has been shown that acute (i.e., endurance exercise) and chronic (i.e., short-term endurance training, long-term combined resistance and endurance training) exercise enhances mucosal immune function, marked by increased salivary SIgA, and offers health benefits in elderly healthy individuals. In contrast, resistance training may not constitute an appropriate method to alter or modify the immune system. Furthermore, regular exercise reduces the risk of chronic metabolic and cardiorespiratory diseases, in part because exercise exerts anti-inflammatory effects (11, 100). For instance, moderate- to high-intensity endurance training (i.e., 12-24 weeks) and strength training (i.e., 24 weeks) created an anti-inflammatory environment and a better inflammatory balance in older people, marked by an increase in IL-10 levels. However, further studies are warranted to address several gaps in knowledge.

## Author Contributions

All authors contributed to the article and approved the submitted version.

## Funding

This study was funded by a EUREP GRANT number UREP26-043-3-018

## Conflict of Interest

The authors declare that the research was conducted in the absence of any commercial or financial relationships that could be construed as a potential conflict of interest.
